# Exposure to Ambient Particulate Matter Induced COPD in a Rat Model and a Description of the Underlying Mechanism

**DOI:** 10.1038/srep45666

**Published:** 2017-03-31

**Authors:** Fang He, Baoling Liao, Jinding Pu, Chenglong Li, Mengning Zheng, Lingmei Huang, Yumin Zhou, Dongxing Zhao, Bing Li, Pixin Ran

**Affiliations:** 1 The State Key Lab of Respiratory Disease, Guangzhou Institute of Respiratory Disease, The First Affiliated Hospital, Guangzhou Medical University, Guangzhou, China; 2 The School of Basic Medicine, Guangzhou Medical University, Guangzhou, China; 3 The Research Center of Experiment Medicine, Guangzhou Medical University, Guangzhou, China

## Abstract

While the health effects of air pollution have been an international public health concern since at least the 1950s, recent research has focused on two broad sources of air pollution, namely, biomass fuel (BMF) and motor vehicle exhaust (MVE). Many studies have shown associations between air pollution PM and exacerbations of pre-existing COPD, but the role of air pollution PM in the development and progression of COPD is still uncertain. The current study indicates that rats can develop pronounced COPD following chronic exposure to air pollution PM (BMF and MVE), as characterized by lung function reduction, mucus metaplasia, lung and systemic inflammation, emphysema, and small airway remodeling. Comparative analyses demonstrate that both BMF and MVE activate similar pathogenesis that are linked to the development of COPD. These findings also show that some differences are found in the lungs of rats exposed to BMF or MVE, which might result in different phenotypes of COPD.

Chronic Obstructive Pulmonary Disease (COPD) is an inflammatory lung disease characterized by progressive and largely irreversible airflow obstruction that involves structural changes in the lung, including emphysema and small airway remodeling^1^,^2^. Although tobacco smoking is the primary cause of COPD, many other environmental and occupational exposures contribute to its pathology. While the health effects of air pollution have been an international public health concern since at least the 1950s, recent research has focused on two broad sources of air pollution, namely, biomass fuel (BMF) and motor vehicle exhaust (MVE)^3^. BMF smoke is now recognized as a major cause of COPD, particularly among women exposed to extremely high concentrations of household air pollution due to the burning of BMFs on inefficient cooking stoves. MVE has acute negative effects on respiratory health^4^,^5^,^6^. A 35-year prospective study of over 57,000 Danes seemed to confirm the previous findings of cross-sectional studies that traffic-related air pollution is a likely cause of COPD^7^.

Inhalable particulate matter (PM), a major component in air pollution, particularly in the fine and ultrafine ranges (diameter <2.5 μm, i.e., PM_2.5_) has been implicated as having a detrimental role in the pathogenesis of COPD. It has been demonstrated that PM has an incremental capacity to penetrate the most distal airway units and potentially into the systemic circulation, as the size of the PM decreases^8^.

Despite its prevalence, the impact of ambient PM on the health of exposed individuals remains poorly understood. Even less is known about the specific processes responsible for the development of pathological and functional changes in the lungs. Through the development of a rat model of air pollution PM exposure, this study sought to better understand the biological consequences that occur during the onset of COPD and the pathogenesis of the disease. To accomplish this, we assessed airway pathologies, pulmonary inflammation, airspace enlargement and pulmonary function in rats exposed to BMF or MVE for up to 7 months, and comparative analyses were conducted between BMF- and MVE-exposed rats to determine what pathogenic mechanisms are linked to the development of COPD^9^.

## Results

### Determination of Particle Size Distributions in Suspension and Gas Concentrations in the Exposure Rooms During Air Pollution PM Exposure

**Figure 1:**
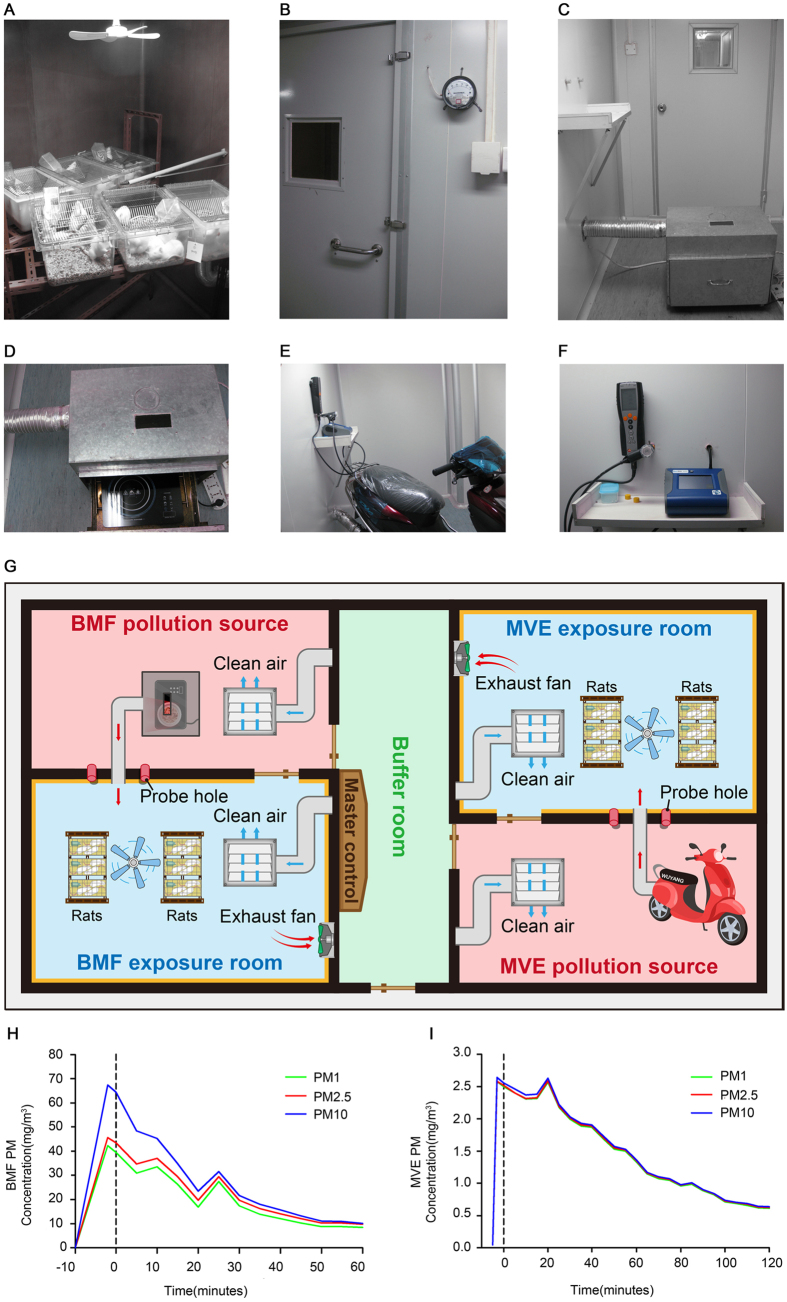
The air pollution PM (BMF and MVE) exposure system. (**A**) The size of the animal exposure room was 3.3 m/L × 2.2 m/W × 2.2 m/H, and the walls were covered with Teflon. Exhaust was directed towards the animal exposure room through a metal tube. Fresh air was sent into the exposure room through an air pump (2.5 L/min) located above the ceiling of the room. A small fan was installed and operated inside the room to improve particle distribution. An exhaust fan connected to a ventilating duct was also installed in the exposure room, enabling rapid discharge of BMF or MVE. (**B**) The pressure inside the animal exposure room was approximately −5 Pa relative to the pressure in the wood-burning/motorcycle room next door. (**C**,**D**) The wood-burning room included a stove (450 w) and a piston pump. (**E**) A Wuyang model WY48QT-2, 1.6 Kw, 125 cc, one-cylinder, four-cycle, gasoline-powered motorcycle was used as a source of MVE. (**F**) PM mass concentration and particle size distribution were measured using a DustTrak (TSI 8533, USA). The NO, NO_x_, SO_2_, CO and O_2_ concentrations were measured using an electrochemical gas analyzer (Testo 340, Germany). (**G**) Schematic illustration of the air pollution PM (BMF and MVE) exposure system. (**H**) The concentrations of PM_2.5_, PM_10_ and PM_1_ were significantly different in the BMF exposure room, with the concentration of PM_10_ being the highest. (**I**) The concentrations of PM_2.5_, PM_10_ and PM_1_ were almost the same in the MVE exposure room.

PM mass concentrations and particle size distributions were measured during air pollution PM exposure. The mean ± SEM value of PM_2.5_, PM_10_ and PM_1_ was 20.406 ± 0.307 mg/m^3^, 24.166 ± 0.526 mg/m^3^, and 18.41 ± 0.311 mg/m^3^ in the BMF exposure room, respectively. In the MVE exposure room, the mean ± SEM value of PM_2.5_, PM_10_ and PM_1_ was 1.46 ± 0.034 mg/m^3^, 1.47 ± 0.034 mg/m^3^, and 1.45 ± 0.035 mg/m^3^, respectively. The variations from each exposure in both the BMF exposure room and the MVE exposure room are depicted in Fig. 1 and Table 1. The concentrations of PM_2.5_, PM_10_ and PM_1_ were significantly different in the BMF group, and the concentration of PM_10_ was the highest. In contrast, the concentrations of PM_2.5_, PM_10_ and PM_1_ were nearly the same in the MVE group.

We also measured gas concentrations inside the chambers because gaseous co-pollutants are generated by combustion. The O_2_, CO, NO_X_ and SO_2_ levels in the exposure rooms during BMF and MVE exposure are shown in Table 2 and Supplementary Fig. S1. The CO and NO_X_ levels were higher in the BMF group than in the MVE group, whereas the O_2_ levels were not significantly different between the two groups, and the SO_2_ levels were low in both groups^10^.

### Air Pollution PM Induces Pulmonary Inflammation

**Figure 2:**
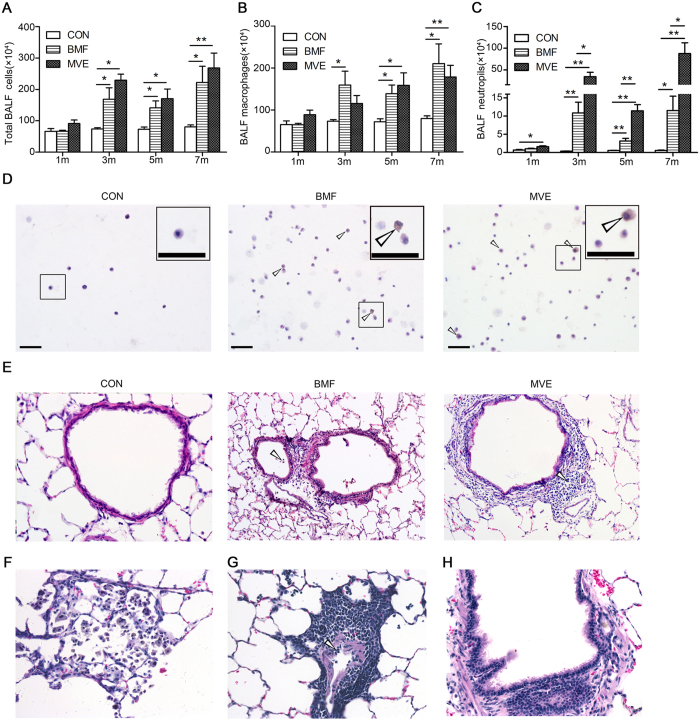
Air pollution PM induces pulmonary inflammation. Air pollution PM exposure increases bronchoalveolar lavage (BAL) leukocyte counts in rats. Rats were exposed to BMF, MVE or clean air (n = 4–6) for 1, 3, 5, and 7 months, and BAL was performed at each time point. Total and differential leukocytes were counted in the BALF samples. (**A**) BALF total leukocyte counts. (**B**) BALF macrophage counts. (**C**) BALF polymorphonuclear neutrophil (PMN) counts. As with the rats exposed to BMF, the MVE-exposed rats showed increased PMN counts, but the macrophage counts in the BALF samples from the BMF-exposed rats were significantly increased compared to those in the MVE-exposed rats. (**D**) Cells in BALF were stained with H&E. Significant increases in BAL PMN counts were detected in the BMF- and the MVE-exposed groups compared to the controls, and the presence of particle-containing macrophages in BALF was observed in the BMF group and the MVE group compared to the controls at 7 months into the exposure period (n = 6). The white arrow indicates a particle-containing macrophage. (**E**) Lung sections stained for H&E from rats exposed to BMF, MVE or clean air (n = 4) for 3 months. The white arrow indicates inflammatory cell infiltrate in the bronchial lumen and the airway walls. (**F**) Small foci of macrophage accumulation occurred in the alveolar areas of the rats as early as 3 months into the exposure period (n = 4). (**G**,**H**) Rats exposed to BMF or MVE for 3 months developed increased numbers of lymphoid aggregates in their lung parenchyma and around their pulmonary vessels and airways (n = 4). Data are expressed as the means ± SEM (**A**–**C**) *P < 0.05, **P < 0.01. Original magnification, x400 (**D**), x200 (**E**–**H**).

The rats exposed to BMF or MVE showed increased total leukocyte counts in the BALF samples compared to the controls as early as 1 month into the exposure period, and these remained elevated after 7 months of BMF and MVE exposure (Fig. 2A). The increases in BALF leukocyte counts were due mainly to increases in macrophage counts. Significant increases in BALF macrophage counts and PMN counts were detected after 7 months of BMF or MVE exposure (Fig. 2B,C). As with the rats exposed to BMF, the MVE-exposed rats showed increased PMN counts in the BALF, whereas the macrophage counts in the BMF-exposed rats were significantly higher than those in the MVE-exposed rats. We also observed the presence of particle-containing macrophages in the BALF from the exposure groups (Fig. 2D).

Examination of H&E-stained lung sections from the exposure groups revealed infiltration of inflammatory cells into the airway walls, the lumen and the distal lung parenchyma (Fig. 2E). Lung inflammation in the MVE-exposed rats was observed earlier and was more severe than that in the BMF-exposed rats. Mild foci of macrophage accumulation occurred in the alveolar areas of the rats as early as 3 months after PM exposure (Fig. 2F).

Rats exposed to BMF or MVE for 3 months developed increased numbers of lymphoid aggregates in lung parenchyma and around pulmonary vessels and airways (Fig. 2G,H). These results are consistent with observations in human patients who develop COPD, in which an adaptive immune response characterized by the formation of lymphoid follicles and aggregates around the airways and in the lung parenchyma is common.

### Air Pollution PM Induces Emphysematous Changes and Airway Remodeling

**Figure 3:**
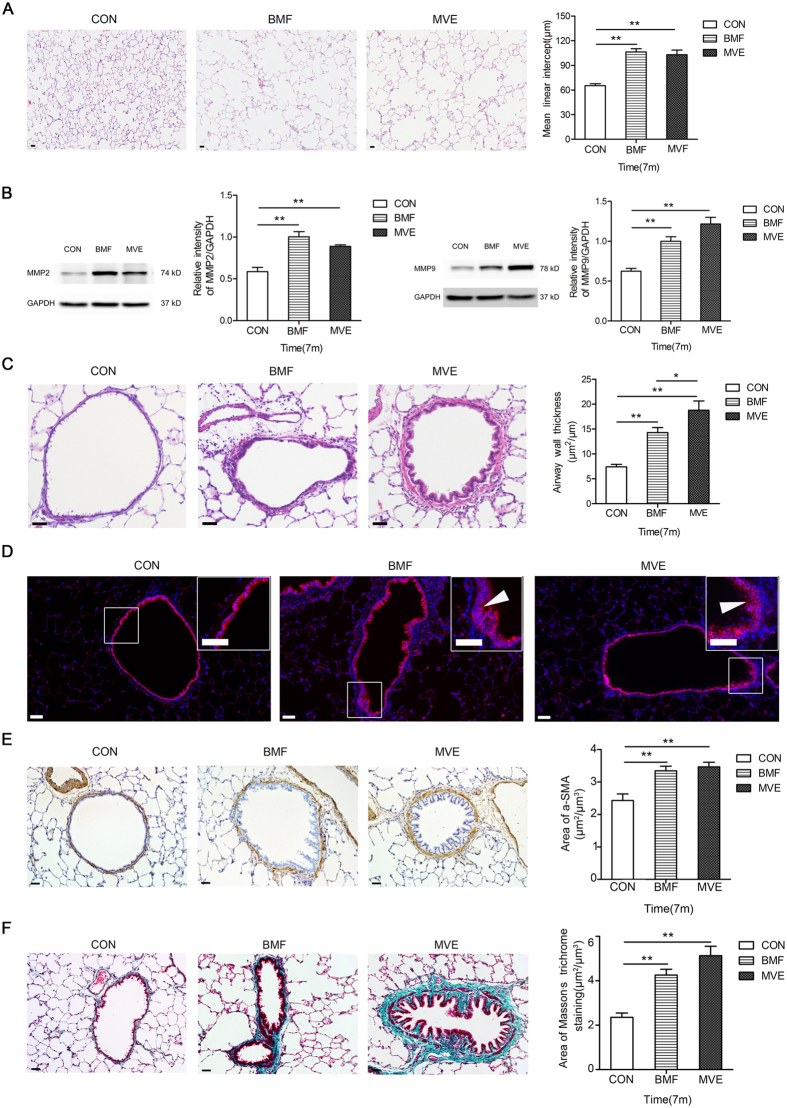
Air pollution PM induces emphysematous changes and airway remodeling. (**A**) Lung sections stained for H&E. Rats developed significant airspace enlargement after 7 months of air pollution PM exposure (BMF and MVE) (n = 8). (**B**) Western blots of lung sections showed that the expression levels of MMP9 and MMP2 (GAPDH was used as a loading control) in the BMF group and the MVE group were markedly increased in comparison with the controls (n = 8). (**C**) Lung sections stained for H&E showed that the thickness of the small airway wall increased significantly in the rats after 7 months of air pollution PM exposure (BMF and MVE) (n = 8). (**D**) Photomicrographs showing significant differences in small airway immunostaining for E-cadherin as well as in the thickness of the bronchiolar epithelium in the BMF group and the MVE group compared to the controls (n = 8). (**E**) Lung sections were immunostained with airway smooth muscle marker (a-SMA). The stained area of smooth muscle around the small airway wall was significantly increased after 7 months of air pollution PM exposure (BMF and MVE) (n = 8). (**F**) Masson’s trichrome staining for type I collagen showed significantly increased collagen deposition in the small airway wall after 7 months of BMF or MVE exposure compared to the controls (n = 8); the deposition of collagen around airways was more severe in the lungs of the rats exposed to MVE than those exposed to BMF. Data are shown as the mean ± SEM *P < 0.05, **P < 0.01. Original magnification, x200 (**C,E,F**) and x100 (**A,D**).

Long-term PM exposure for 3, 5, and 7 months damaged the lung parenchyma, which caused modifications in the distal airspaces, resulting in alveolar enlargement. Histological analysis revealed the presence of emphysematous lesions at 7 months, and the mean linear intercept difference was significant between the controls and the exposure groups (Fig. 3A). Carlos Ramos reported that increases in MMP9 and MMP2 activity and expression were responsible for the induction of emphysematous lesions following wood smoke exposure in guinea pigs^11^. Western blotting revealed that the expression levels of MMP9 and MMP2 in the BMF group and the MVE group were markedly increased in comparison with the controls (Fig. 3B). Walter A and colleagues found diesel (DIE)-exposed animals showed higher levels of apoptosis in lung sections evaluated using TUNEL staining^12^. The present study revealed that significant increases in apoptosis were detected in lung tissues after 3 and 5 months of BMF or MVE exposure and that the apoptosis of alveolar septal cells was more severe in the rats exposed to BMF (Supplementary Fig. S3).

At 7 months into the exposure period, the thicknesses of the small airway walls (Pbm of airways less than 2000 μm) in the exposure groups were markedly increased in comparison with the controls (Fig. 3C). Additionally, the average small airway wall thickness in the MVE-exposed rats was significantly increased compared to that in the BMF-exposed rats.

After 7 months of air pollution PM exposure, significant differences were observed in bronchiolar epithelium thickness in the exposure groups compared to the control group. Microscopic examination of the lungs (7 rats among 8 rats) revealed epithelial cell proliferation in the lungs of the BMF-exposed rats, which was more obvious than that found in the lungs of the MVE-exposed rats (4 rats among 8 rats) (Fig. 3D).

Airway smooth muscle thickness was quantified based on the area of a-SMA IHC staining. Both BMF and MVE exposure induced significant increases in small airway smooth muscle thickness after 7 months of exposure (p < 0.05) (Fig. 3E). Masson’s trichrome staining of lung sections revealed increased deposition of collagen around the small airways in the lungs of the BMF group and the MVE group at 3, 5, and 7 months (Fig. 3F). The deposition of collagen around airways was more severe in the rats exposed to MVE.

### Air Pollution PM Exposure Induces Mucus Metaplasia in the Large and Small Airways of Rats

**Figure 4:**
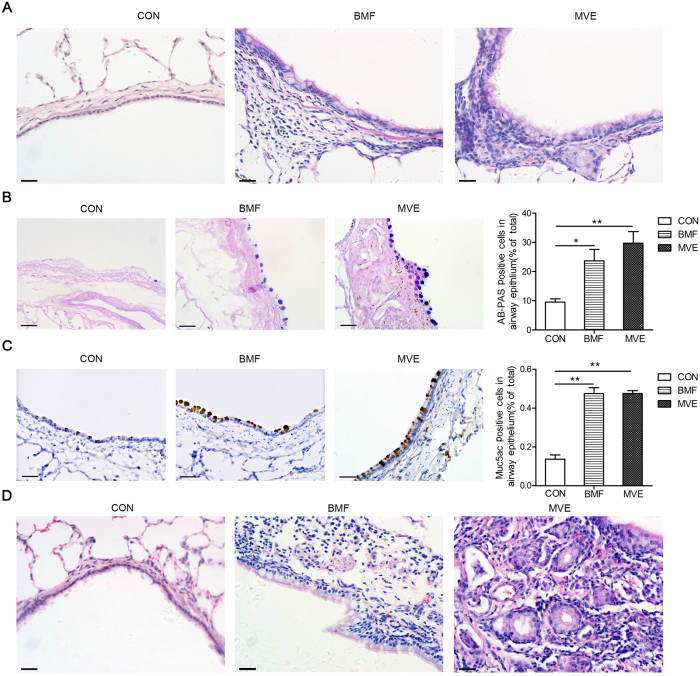
Air pollution PM exposure induces mucus metaplasia in the large and small airways of rats. (**A**) Lung sections stained for H&E showed obvious increases in goblet cell number after 3 months of air pollution PM exposure (BMF and MVE) (n = 4). (**B**) Air pollution PM exposure induced mucus metaplasia in large and small airways. Lung sections from rats exposed to BMF, MVE or clean air for 3 months were stained with AB-PAS (n = 4). (**C**) Lung sections were immunostained with MUC5AC. The expression levels of MUC5AC were markedly increased in the BMF group and the MVE group compared to the controls at 3 months into the exposure period (n = 4). (**D**) Representative images of airway submucosal glands in air pollution PM-exposed rats. Lung sections stained for H&E showed that bronchial submucosal glands could not be visualized in the control group, but hyperplasia of submucosal glands was easily identifiable in the BMF group and the MVE group at 5 months into the exposure period (n = 4). Original magnification, x400 (**A–D**).

H&E-stained lung sections (Fig. 4A) revealed clear increases in goblet cell populations after 3 months of exposure. Lung sections stained with AB-PAS stain (Fig. 4B) or immunostained for MUC5AC (Fig. 4C) revealed that the rats in the control group had few airway mucin-expressing cells, whereas mucus cell metaplasia was readily evident in the airway epithelia of the rats in the exposure groups at 3 months and increased further after 5 months of PM exposure. Mucus cell metaplasia was worse in the rats exposed to MVE than in those exposed to BMF in the epithelia of both large and small airways (Fig. 4B,C). In contrast to humans, rats do not have prominent bronchial submucosal glands. We could not identify bronchial submucosal glands in the control group, whereas the rats in the BMF group and the MVE group exposed to air pollution PM for 5 months showed submucosal gland hyperplasia and hypertrophy in their large airways (Fig. 4D).

### Air pollution PM Exposure Induces Epithelial-mesenchymal Transition in Airway Epithelial Cells, Leading to Airway Remodeling

**Figure 5:**
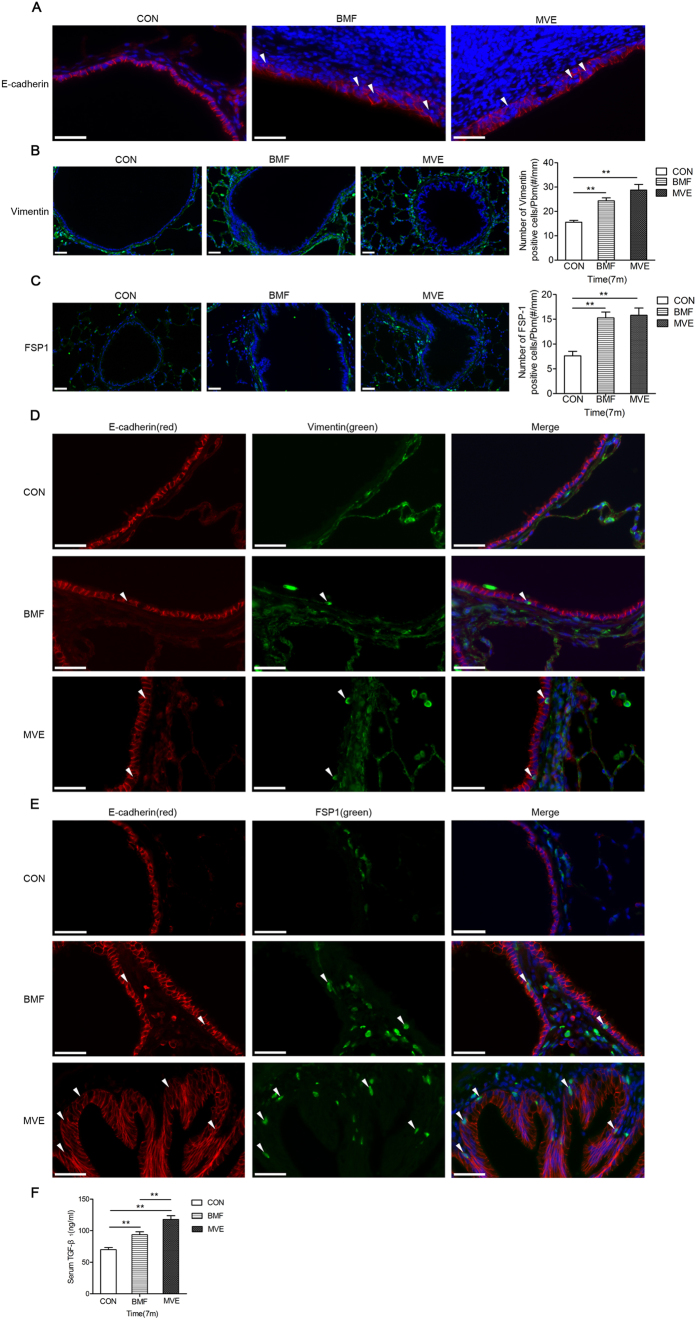
Air pollution PM exposure induces airway epithelia epithelial-mesenchymal transition (EMT). (**A**) Photomicrographs showing small airways immunostained for E-cadherin. The airway epithelia in the rats exposed to air pollution PM showed a decrease in E-cadherin immunostaining (BMF and MVE group) compared to the controls after 7 months of exposure (n = 8). (**B,C**) Immunofluorescent staining showed positive staining of mesenchymal markers (vimentin and FSP1) was significantly increased in the small airway and could be observed in small airway epithelia of the rats exposed to BMF or MVE for 7 months (n = 8). (**D,E**) Photomicrographs showing the small number of cells that were double-immunostained for E-cadherin and Vimentin or FSP1 in the airway epithelia of the rats exposed to BMF or MVE for 7 months (n = 8). The white arrows show positively immunostained cells. (**F**) ELISA results showed a significant increase in the concentration of TGF-β1 in serum from the BMF- and MVE-exposed rats after 7 months of exposure (n = 8). The concentration of TGF-β1 in the MVE-exposed rats was significantly higher than that in the BMF-exposed rats. Data are shown as the mean ± SEM (**B, C, F**) *P < 0.05, **P < 0.01. Original magnification, x400 (**A,D,E**) and x200 (**B,C).**

Epithelial-mesenchymal transition (EMT) is a process in which epithelial cells undergo a transition to a motile mesenchymal phenotype^13^. It can be considered a marker of profound epithelial plasticity^14^. The airway epithelia in the rats exposed to BMF or MVE showed decreases in E-cadherin immunostaining compared with those in the controls (Fig. 5A). Additionally, positive staining for mesenchymal markers (FSP1 and vimentin) was significantly increased in the exposure groups compared to that in the control group and could be observed in small airway epithelia at 3, 5 and 7 months (Fig. 5B,C). Positive staining for mesenchymal markers in the airway was higher in the rats exposed to MVE than in those exposed to BMF. Furthermore, Fig. 5D and E show the small number of cells that were double immunostained for E-cadherin and vimentin or FSP1 in the airway subepithelia of the rats in the exposure groups at 7 months. The number of double-stained cells was higher in the MVE group than in the BMF group. Recently, it was demonstrated that TGF-β_1_ could induce EMT in alveolar epithelial cells *in vitro* and *in vivo*^15^, as well as in human bronchial epithelial cells^16^. At the end of the experiment, after 7 months of exposure, both the BMF- and MVE-exposed rats had significant increases in serum concentrations of TGF-β1 compared to the controls. Furthermore, the average concentration of TGF-β1 in the MVE group was markedly increased compared to that in the BMF group (Fig. 5F). These data demonstrate that MVE has a stronger effect on airway epithelia EMT than BMF.

### Effects of Air Pollution PM on Rat PFT Results

**Figure 6:**
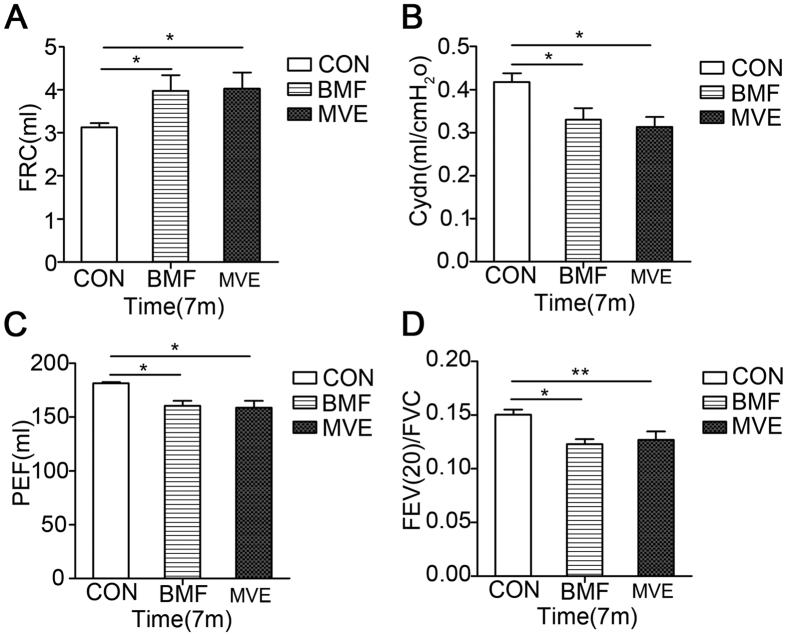
Effects of air pollution PM on Rat PFT results. After 7 months of exposure, PEF, FEV(20)/FVC and Cdyn (**C,D**, and **B**) were significantly lower in the BMF-exposed rats (n = 6) and the MVE-exposed rats (n = 6) versus the control rats (n = 6), while FRC (**A**) was significantly higher. Data are expressed as the mean ± SEM (**A–D**) *P < 0.05, **P < 0.01.

Exposure of rats to BMF or MVE caused statistically significant effects on pulmonary function test (PFT) results during the 7-month study period. Both BMF and MVE exposure affected functional residual capacity (FRC), dynamic pulmonary compliance (Cdyn), peak expiratory flow (PEF), and forced expiratory volume at 20 ms/forced vital capacity (FEV(20)/FVC) (Fig. 6A,B,C and D). After 7 months of exposure, the FEV(20)/FVC in the exposure groups showed a significant decreasing trend compared to that in the control group (p < 0.05). Additionally, the rats exposed to BMF or MVE for 7 months had increases in FRC and decreases in Cdyn and PEF. These data show that exposure to BMF or MVE reduced lung function in rats.

### Air Pollution PM Triggered Inflammatory Cytokine Expression and Induced Systemic Inflammatory Responses

Airway epithelial cells serve as a barrier to noxious stimuli and produce mediators and enzymes to maintain normal airway homeostasis. These cells can also serve as effectors to initiate and orchestrate immune and inflammatory responses by releasing chemokines and cytokines, which regulate monocyte/macrophage and neutrophil accumulation and subsequent lung pathology^17^. Because we observed significant accumulation of inflammatory cells in the lungs of the experimental rats, we characterized the inflammatory milieu by measuring key cytokines and chemokines in BALF and serum using a bead-based multiplex assay.

**Figure 7:**
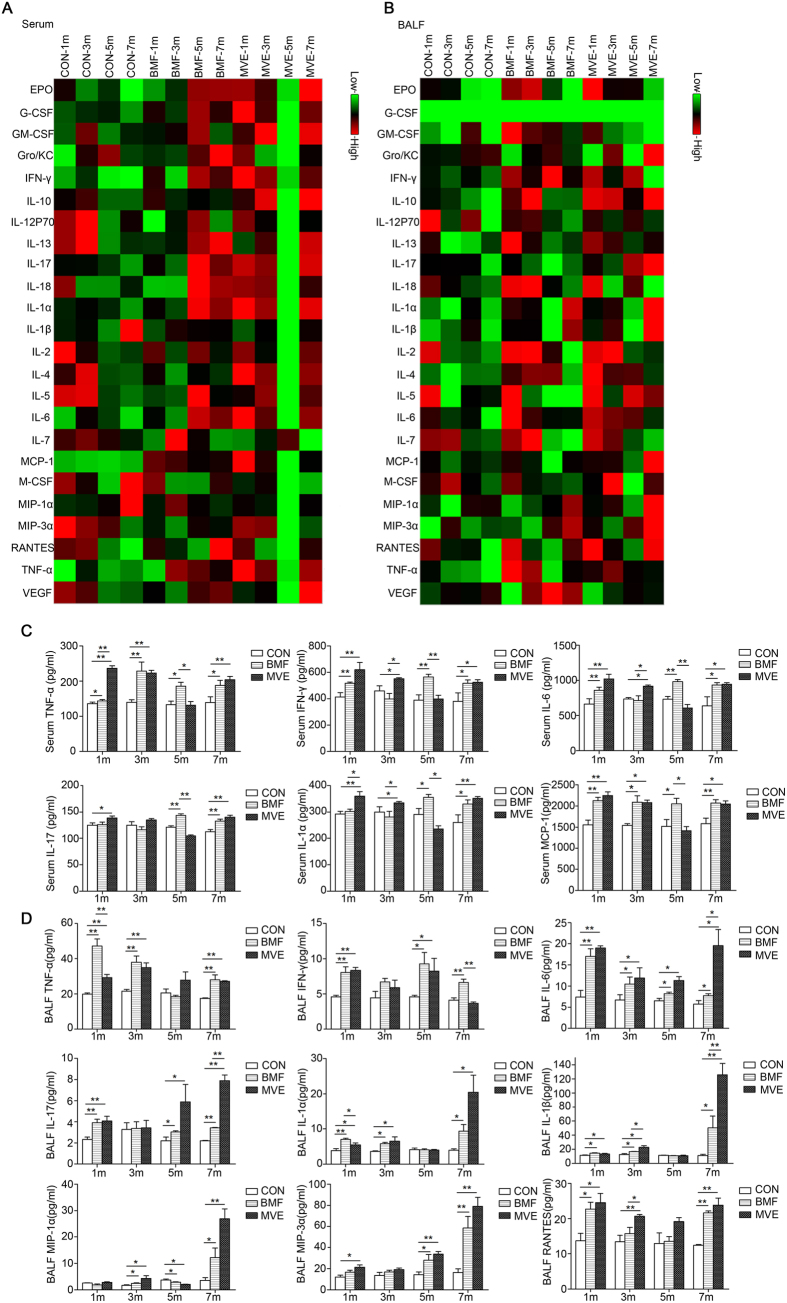
Air pollution PM induces expression of inflammatory cytokines. BALF and serum samples from rats were assayed for cytokine expression as described in the METHODS section. Raw data were analyzed using a Bio-Plex system (Bio-Rad, CA, USA). (**A**) (serum), (**B**) (BALF): The means of the raw data for each group were z-score-transformed and presented as a heatmap using DataPro software. The transformation was performed for each analyte individually. Data representative of three experiments. N = 4–6 rats per group. (**C**) (serum), (**D**) (BALF): The expression of inflammatory cytokines significantly changed in the serum and BALF after 1, 3, 5 and 7 months of air pollution PM exposure compared to clean air exposure. G-CSF = granulocyte colony-stimulating factor; GM-CSF = granulocyte-macrophage colony-stimulating factor; M-CSF = macrophage colony-stimulating factor; MCP = monocyte chemoattractant protein; MIP = macrophage inflammatory protein; RANTES = regulated upon activation, normal T-cell expressed and secreted; TNF = tumor necrosis factor; EPO = erythropoietin, Gro/KC = chemokine (C-X-C motif) ligand 1 (CXCL1), VEGF = vascular endothelial growth factor.

Elevated cytokine levels were found in the sera and BALF of the BMF- or MVE-exposed rats after 1, 3, 5, and 7 months (Fig. 7A,B). In total, 8 cytokines out of the 24 tested in serum and 9 out of the 24 tested in BALF exhibited significant increases in expression (p < 0.05 vs. control) after 1 month of exposure to MVE. The following cytokines showed concentration changes in serum: EPO, IL-17, MCP-1, IL-6, G-CSF, IL-1α, IFN-γ, and TNF-α. The following cytokines showed changes in BALF: IL-17, MIP-3α, RANTES, IL-1α, IL-1β, IFN-γ, IL-6, IL-4, and TNF-α. After 7 months of exposure to MVE, 8 cytokines out of the 24 tested in serum and 9 out of the 24 tested in BALF exhibited significant increases in expression (p < 0.05 vs. control). The changes in cytokine levels in serum that were found after 7 months of exposure to MVE were all lower than those found after 1 month of exposure to MVE. Conversely, in BALF, 5 out of the 9 cytokines measured, including IL-1α, IL-1β, IL-17, MIP-3α and MIP-1α, were significantly up-regulated after 7 months of MVE exposure compared to 1 month of exposure. Furthermore, TNF-α and IFN-γ were down-regulated (Fig. 7C,D).

After 1 month of BMF exposure, a total of 4 cytokines out of the 24 tested in serum and 9 out of the 24 tested in BALF exhibited significant increases in expression (p < 0.05 vs. control). The following cytokines showed concentration changes in serum: MCP-1, IL-6, IFN-γ, and TNF-α. The following cytokines showed concentration changes in BALF: IL-13, IL-17, MCP-1, IL-1α, IL-1β, RANTES, IL-6, IFN-γ, and TNF-α. After 7 months of BMF exposure, a total of 8 cytokines out of the 24 tested in serum and 8 out of the 24 tested in BALF exhibited significant increases compared to those of the controls (p < 0.05 vs. control). The following cytokines showed concentration changes in serum: EPO, IL-17, MCP-1, IL-6, G-CSF, IL-1α, IFN-γ, and TNF-α. The following cytokines showed concentration changes in BALF: IL-17, MIP-3α, IL-1α, IL-1β, RANTES, IL-6, IFN-γ, and TNF-α. The increases in serum cytokine levels following 7 months of exposure to BMF were higher than those observed after 1 month of exposure. In BALF, 4 out of the 8 cytokines measured, including IL-1α, IL-1β, MIP-3α and MIP-1α, were significantly up-regulated after 7 months of exposure compared to after 1 month of exposure, whereas TNF-α, IFN-γ and IL-6 were down-regulated (Fig. 7C,D).

Overall, higher cytokine levels were found in both serum and BALF following exposure to MVE than in those after exposure to BMF (Fig. 7C,D). Among three groups and four time points, almost all cytokines tested in serum were at their highest levels after 1 month of exposure to MVE, and this was consistent with when the highest degree of pulmonary inflammation was observed in the MVE-exposed rats. After 5 months of exposure to BMF, almost all cytokines tested in serum were at their highest levels among the three groups. This was consistent with the systemic inflammatory responses observed in the BMF-exposed rats, which showed the greatest reduction in body weight (Supplementary Fig. S2) and the most severe hair loss at this time point. Together, these data demonstrate that air pollution PM acts as a noxious stimulus to airway cells and that rats exposed to such PM are capable of generating a complex inflammatory cytokine environment that recapitulates the phenotype of COPD.

## Discussion

Many studies have shown associations between air pollution and exacerbations of pre-existing COPD, but the role of air pollution in the development and progression of COPD is still uncertain^18^,^19^. It is possible that particle retention in lung tissue and long exposure periods have a more significant impact on lung mechanics or remodeling^20^.

We performed an *in vivo* experiment to determine whether exposing rats to BMF or MVE for up to 7 months results in pronounced COPD. To accomplish this, physical examinations, PFTs, and analyses of BALF and blood serum were performed after 1, 3, 5 and 7 months of exposure to BMF or MVE. The PM mass concentrations of BMF or MVE are according to the upper bound of the indoor air pollution data measured in the kitchen during cooking in rural areas of Southern China^21^,^22^,^23^, and the heaviest haze pollution data measured in Northern China (https://www.aqistudy.cn/). Because of the “real world exposure” approach employed in this study, we can include the possibility that gases and some other ambient factors such as temperature and humidity could have influenced our results. The rats in our study were exposed to BMF or MVE for 1, 3, 5 and 7 months, a strategy that may be useful for serial sampling for COPD biomarker studies. Our study showed, for the first time, that a rat model of air pollution PM-induced COPD, especially MVE-induced COPD, can be successfully established and that air pollution PM-exposed rats develop changes in their airways that are consistent with those occurring in human COPD patients. These changes included the following: lung inflammation, emphysema, small airway remodeling, airway mucus hypersecretion, lung function reduction and systemic inflammatory response.

The pulmonary airways form the first line of defense against airborne irritants, pollutants, and other infectious agents. In addition to providing a mechanical barrier, airway epithelia also produce chemokines and cytokines that recruit and activate phagocytic cells to clear inhaled particles/agents and damaged cells. An increase in phagocytic inflammatory cell number and elevated protein levels in BALF upon exposure to air pollution PM was previously reported^24^,^25^,^26^. Similar observations were made in our current study, in which air pollution PM induced the accumulation of PMNs/leukocytes, followed by an influx of AMs (Fig. 2B,C). This is also in agreement with previous studies reporting increased numbers of macrophages and enhanced presence of particle-containing AMs in lungs following chronic inhalation exposure to BMF or MVE (Fig. 2D,F). These data demonstrate that inhaled PM is characterized by impaired clearance or prolonged retention, which can further lead to respiratory injury.

In our study, the rats in both the BMF group and the MVE group showed characteristic pathological changes consistent with those that occur in human COPD patients, which supported the hypothesis that MVE exposure induced COPD. We also compared the histopathological changes that were produced in the lungs of the rats exposed to BMF or MVE and found some differences (Figs 2,3 and 4). We found that MVE exposure causes obvious neutrophilic airway inflammation, while BMF exposure causes a prominent increase in the presence of phagocytic inflammatory cells, primarily macrophages. The rats exposed to MVE had earlier and more severe lung inflammation, greater numbers of goblet epithelial cells and thicker small airway walls with collagen deposition, whereas the rats exposed to BMF had a higher incidence of emphysema, more severe apoptosis of alveolar septal cells (Supplementary Fig. S3), greater epithelial cell hyperplasia and enhanced squamous metaplasia. These results conclusively show that both BMF and MVE exposure could activate similar pathogenesis linked to the development of COPD. Different sources of PM, such as MVE or BMF, might induce some different pathological changes and result in different phenotypes of COPD. Mauderly JL reported that engine exhaust arguably elicits a greater response than wood smoke, which is partially consistent with the present finding that the rats exposed to MVE had earlier and more severe lung inflammation; however, in contrast to our findings of inducing pronounced COPD in rats, Mauderly JL found no exposure (engine exhaust or wood smoke) caused overt illness and histopathology of major organs visible by light microscopy^27^. The rats in our study were exposed to BMF or MVE for higher PM concentrations and longer exposure periods, which may explain the differences between the findings of the two studies.

A novel explanation for the mechanism underlying the remodeling that occurs in inflammatory respiratory disease, which may be applicable to COPD, involves the differentiation of airway epithelial cells to a mesenchymal phenotype, with subsequent migration through the reticular basement membrane (Rbm) to the subepithelial lamina propria, a process known as EMT^28^. We also found that exposure to air pollution PM was involved in increased EMT, as shown by increases in mesenchymal markers, cell contractility, and TGF-β activation. In rats exposed to BMF or MVE for 7 months, positive staining of mesenchymal markers (FSP1 and vimentin) increased and could be observed in the airway epithelium. Additionally, immunofluorescent staining showed that the expression of E-cadherin had decreased. Furthermore, cells that were double immunostained for E-cadherin and vimentin or FSP1 were observed in the airway subepithelium (Fig. 5). The concentration of TGF-β1 in serum significantly increased after 7 months of exposure to BMF or MVE. Our findings showed that MVE exposure has a stronger effect on airway epithelial cell EMT than BMF exposure. Bronchial epithelial cell EMT plays a significant role in airway tissue remodeling and peri-bronchial fibrosis in the pathogenesis of lung disease^29^.

Airway obstruction is thought to mainly occur in the small airways, but the pathology affects the whole bronchial tree^30^. Airway remodeling in COPD is mostly related to reduced airflow due to small airway fibrosis and, ultimately, obliteration. We evaluated lung function in rats after 7 months of exposure to BMF or MVE (Fig. 6). In both experimental groups, significant reductions in lung function were observed, manifested by increased resistance and FRC and decreased Cdyn, PEF and FEV(20)/FVC.

As key events in COPD, pulmonary inflammation and systemic inflammation are highly associated with the incidence and progression of the disease^31^. The injurious effects of ambient PM are both local (in the lung) and systemic. PM contributes to the systemic inflammatory response and the functional alteration of multiple organs^32^, mainly through activation of inflammatory cells and related inflammatory mediators that infiltrate into systemic circulation through lung tissue.

Cytokines play a key role in orchestrating chronic inflammation and structural changes in the respiratory tract in COPD by recruiting, activating, and promoting the survival of multiple inflammatory cells^33^,^34^. To the best of our knowledge, this study reports a much broader assessment of inflammatory mediators in serial rat BALF and serum samples after air pollution PM (BMF and MVE) exposure (Fig. 7). The majority of inflammatory mediators tested, including IL-13, IL-17, MIP-3α, MIP-1α, MCP-1, IL-1α, IL-1β, RANTES, IL-6, TNF-α and IFN-γ, were up-regulated in BALF and serum upon MVE or BMF exposure, which is consistent with the recruitment of phagocytic cells, such as neutrophils and macrophages, that was observed (Fig. 2). The accumulation of proinflammatory cytokines, such as TNF-α, IL-1α, and IL-6, was found upon stimulation of epithelial cells with air pollution PM. Furthermore, an incremental change in the release of chemokines, notably RANTES, MCP-1 and MIP-1α, was produced by activated macrophages and epithelial cells. In particular, the prolonged accumulation of IL-1α, IL-1β, MCP-1 and MIP-1α further supported the persistent presence of macrophages in the BALF and pigment-laden macrophages in the lungs of the BMF and MVE groups (Fig. 2). As a multifunctional growth factor, the concentrations of TGF-β1 in serum were significantly increased in the BMF- or MVE-exposed rats after 7 months of exposure. These changes resulting from air pollution PM exposure are capable of triggering long-term adverse effects in the lung.

**Figure 8:**
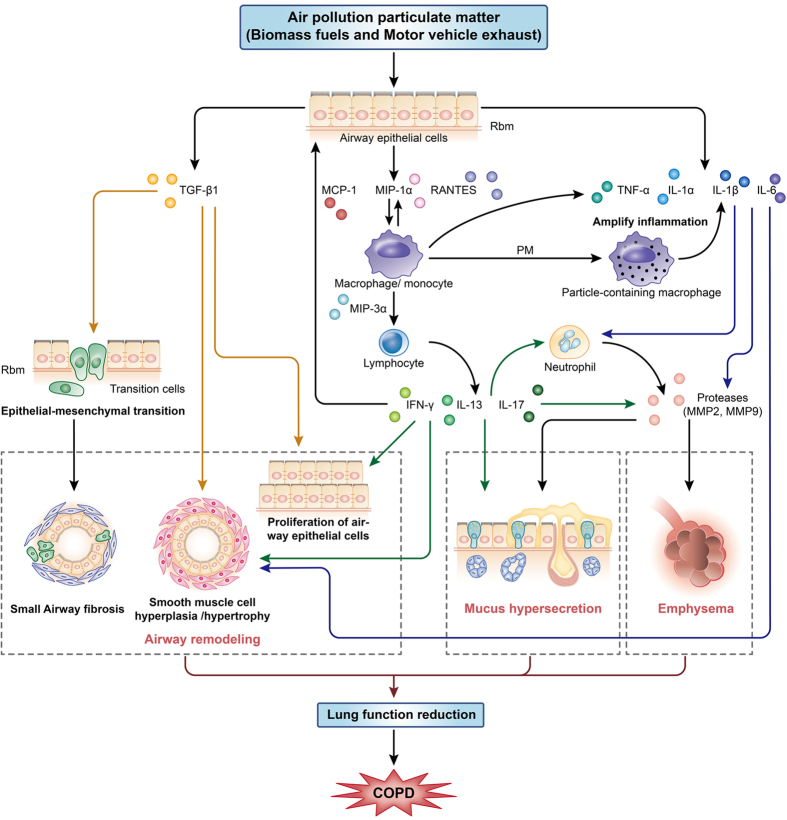
A schematic depicting how exposure to air pollution PM (BMF and MVE) induced COPD in a rat model. Air pollution PM can activate airway epithelial cells and macrophages to release multiple cytokines, including the growth factor TGF-β1, which stimulates EMT and fibroblast proliferation, resulting in small airway remodeling. These cells also secrete several chemokines, such as MCP-1, MIP-1α, MIP-3α and RANTES, which attract circulating cells into the lungs in addition to proinflammatory cytokines, such as TNF-α, IL-1β, and IL-6, which amplify inflammation and can lead to elastin degradation and alveolar destruction. These events eventually cause functional changes in the lungs, especially lung function reduction. Air pollution PM exposure causes airway cells to release multiple cytokines capable of inducing pronounced COPD in rats.

The results of our study are in agreement with the proposed roles of these cytokines in the pathogenesis of COPD. Inhaled air pollution PM activates epithelial cells and macrophages to release multiple cytokines, including the growth factor TGF-β1, which stimulates EMT and fibroblast proliferation, resulting in small airway remodeling. These cells also secrete several chemokines, such as MCP-1, MIP-1α, MIP-3α and RANTES, which attract circulating cells into the lungs in addition to proinflammatory cytokines, such as TNF-α, IL-1β, and IL-6, which amplify inflammation and can lead to elastin degradation and alveolar destruction^35^. These events eventually increase disease severity and cause functional changes in the lungs, especially lung function reduction (Fig. 8).

## Conclusions

Our current study indicates that both BMF and MVE exposure cause airway cells to release multiple cytokines capable of inducing pronounced COPD in rats. Comparative analyses demonstrated that some differences are found in the lungs of rats exposed to BMF or MVE, which might result in different phenotypes of COPD.

## Methods

### Animals

96 female Sprague-Dawley rats (body weight 180–200 g, 6–8 weeks old) were socially housed (up to four rats per cage) in the laboratory animal center of Guangzhou Medical University. The rats were randomly divided into a BMF group, a MVE group and a clean air control group. The care and use of the animals was in compliance with regulations designated by the Chinese Association for Laboratory Animal Science Policy. All of the experimental protocols were approved by the Institutional Animal Care and Use Committee of Guangzhou Medical University. All animals studied were female because other studies have found that women who smoke have a higher risk of developing COPD than men regardless of smoking level or intensity^36^,^37^.

The animal facility maintained a 12-hour light/dark cycle. The rats were exposed to 100% freshly filtered air with 10 to 15 air changes per hour in the control and exposure groups before initiating the exposures. Rooms were maintained at 20 ± 2 °C with 45–65% relative humidity according to the Guide for the Care and Use of Laboratory Animals of Guangzhou Medical University.

The rats in the BMF group were exposed to PM for four 1-h periods (from 8:00 a.m. to 9:00 a.m., from 10:00 a.m. to 11:00 a.m., from 14:00 p.m. to 15:00 p.m., and from 16:00 p.m. to 17:00 p.m.), 5 days per week, for 1, 3, 5, and 7 months. The control group was composed of 32 rats that were exposed to clean air every day for 7 months. The rats in the MVE group were exposed to PM for two 2-h exposure periods (from 9:00 a.m. to 11:00 a.m. and from 15:00 p.m. to 17:00 p.m.), 5 days per week, for 1, 3, 5, and 7 months. After each period of exposure, the animal exposure room was set to cycle with freshly filtered air for 30 minutes (during which room air was delivered to return the room to its initial conditions). The rats were observed for any sign of illness a minimum of twice daily. The rats were fed twice daily, and water was available at all times. All rats were weighed weekly on an analytical scale. For standardization purposes, all rats were weighed on the same day of the week and at the same time of day.

### Exposure System

*Exposure to BMF*. Rats were exposed to smoke produced by smoldering China fir sawdust (40 g/per time) for four 1-h periods, 5 days per week, for 1, 3, 5, and 7 months. The BMF exposure system primarily consisted of a wood-burning room and a rat exposure room (Fig. 1A,C,G). The size of the animal exposure room was 3.3 m/L × 2.2 m/W × 2.2 m/H, and the walls were covered with Teflon. The BMF generated by the burning stove (450 w) and was sent into the animal exposure room through a piston pump (5 L/min) (Fig. 1C,D). Fresh air was sent into the animal exposure room through an air pump (2.5 L/min) located above the ceiling of the room. During the exposure periods, the entrance of the room was sealed to ensure the air tightness of the room, and a small fan was installed and operated inside the room to improve the distribution of the particles (i.e., mixing). An exhaust fan connected to a ventilating duct was also installed in the exposure room, which enabled the rapid discharge of BMF. In addition to inlet and outlet ports for BMF, there were 2 sampling ports available to monitor various characteristics of the exposure PM and gas. During the exposure periods, the pressure inside the exposure room was maintained at approximately −5 Pa relative to the pressure in the wood-burning room (Fig. 1B).

*Exposure to MVE.* A Wuyang model WY48QT-2, 1.6 Kw, 125 cc, one-cylinder, four-cycle, gasoline-powered motorcycle was used as a source of MVE. The rats exposed to MVE for two 2-h periods, 5 days per week, for 1, 3, 5, and 7 months. The motorcycle was located next door to the rat exposure room, and the MVE was directed towards the exposure room through a metal tube (Fig. 1A,E,G). Fresh air was sent into the exposure room through an air pump (2.5 L/min) located above the ceiling of the room. The motorcycle was operated using premium low-sulfur gasoline (<150 ppm; Petro Inc.) and 5 W-50 motor oil (Mobil SN).

The size of the rat exposure room was 3.3 m/L × 2.2 m/W × 2.2 m/H, and the walls were covered with Teflon. Before each animal exposure session, the motorcycle engine was operated in an idle state for 2 minutes and then stopped for 10 minutes to achieve a stable mass concentration. During the exposure periods, the entrance of the room was sealed to ensure the air tightness of the room. A small fan was installed and operated inside the room to improve particle distribution (i.e., mixing). An exhaust fan connected to a ventilating duct was also installed in the exposure room, enabling the rapid discharge of MVE. In addition to inlet and outlet ports for the MVE, there were 2 sampling ports available to monitor various characteristics of the exposure PM and gas. During the exposure periods, the pressure inside the exposure room was approximately −5 Pa relative to the pressure in the room that housed the motorcycle (Fig. 1B).

### Time Points for Serial Sampling

Two different air pollution PM exposure studies were performed. Rats were exposed to either BMF (n = 32) or clean air (n = 32) for 1 h, 4 times per day, 5 days per week, for 1, 3, 5, and 7 months. Rats were exposed to either MVE (n = 32) or clean air (n = 32) for 2 h, 2 times per day, 5 days per week, for 1, 3, 5, and 7 months. To determine whether airway or airspace pathologies developed in the rats, physical examination and bronchoalveolar lavage fluid (BALF) and blood serum analyses were performed after BMF or MVE exposure for 1, 3, 5, and 7 months. Additionally, lung tissue was obtained at necropsy, and PFTs were performed after air pollution PM exposure for 7 months.

### Lung Function Tests

Rat lung function was tested using a Forced Pulmonary Maneuver System (Buxco Research Systems, Wilmington, NC, USA) following the manufacturer’s protocols^38^. Briefly, rats were anesthetized by intraperitoneal injection of 3% pentobarbital (1 ml/Kg), tracheotomized and intubated, and placed supine in the body chamber of the system. The anesthesia was maintained at a light surgical plane for the duration of testing. The endotracheal tube was attached to an airway port, and the distal end of the catheter was passed through a small opening located near the airway port. The catheter was then connected to the system. The average breathing frequency was set to 70 breaths/min. To measure the FRC, Cdyn, PEF, and FEV(20)/FVC, a quasi-static pressure volume maneuver was performed, which inflates the lungs to a standard pressure of +30 cm H_2_O and then slowly exhales until a negative pressure of −30 cm H_2_O is reached. The quasi-static compliance was defined as the volume/pressure ratio at 50% of the expiration. With the fast flow volume maneuver, the lungs were first inflated to +30 cm H_2_O and immediately afterwards connected to a highly negative pressure to enforce expiration until reaching a residual volume at −30 cm H_2_O. FEV at 20 ms were recorded during this maneuver. For each test of every rat, at least three acceptable maneuvers were conducted to obtain a reliable mean for all numeric parameters.

### Statistical Analysis

All data are presented as the mean values ± SEM from at least three independent experiments. Differences between two groups were analyzed using Student’s t-test, and differences between multiple groups were analyzed with ANOVA. All statistical analyses were performed using SPSS 18.0 software (SPSS Inc., Chicago, USA). p < 0.05 was considered statistically significant.

Detailed methods are provided in the online supplement.

## Additional Information

**How to cite this article**: He, F. *et al*. Exposure to Ambient Particulate Matter Induced COPD in a Rat Model and a Description of the Underlying Mechanism. *Sci. Rep.* **7**, 45666; doi: 10.1038/srep45666 (2017).

**Publisher's note:** Springer Nature remains neutral with regard to jurisdictional claims in published maps and institutional affiliations.

## Supplementary Material

Supplemental Text

## Figures and Tables

**Table 1:** The Concentrations of PM (mg/m^3^) inside the Exposure Room (Mean ± SEM).

	CON	BMF	MVE
PM_1_ mass concentration (mg/m^3^)	0.056 ± 0.006	18.41 ± 0.311	1.45 ± 0.035
PM_2.5_ mass concentration (mg/m^3^)	0.056 ± 0.006	20.40 ± 0.307	1.46 ± 0.034
PM_10_ mass concentration (mg/m^3^)	0.057 ± 0.007	24.16 ± 0.526	1.47 ± 0.034

CON: the clean air control group; BMF: the biomass fuels exposure group; MVE: the motor vehicle exhaust exposure group.

**Table 2:** CO, SO_2_, NO_X_, O_2_ Levels and Exposure Details during BMF and MVE Exposure (Mean ± SEM).

	CON	BMF	MVE
O_2_(%)	20.98 ± 0.009	20.94 ± 0.005	20.95 ± 0.006
CO (ppm)	—	90.15 ± 3.221	67.524 ± 3.565
NO_1_ (ppm)	—	2.96 ± 0.291	0.50 ± 0.211
NO_X_(ppm)	—	2.96 ± 0.291	0.50 ± 0.211
SO_2_(ppm)	—	0.087 ± 0.043	0.35 ± 0.181
Temperture(°C)	20.68 ± 0.130	20.86 ± 0.289	20.60 ± 1.006
Humidity(%)	60.808 ± 1.830	65.13 ± 2.058	54.80 ± 2.129

CON: the clean air control group; BMF: the biomass fuels exposure group; MVE: the motor vehicle exhaust exposure group.

## References

[b1] Hogg J. C. & Timens W. The pathology of chronic obstructive pulmonary disease. Annu Rev Pathol 4, 435–459 (2009). 18954287 10.1146/annurev.pathol.4.110807.092145

[b2] He F. . The Pro-Proliferative Effects of Nicotine and Its Underlying Mechanism on Rat Airway Smooth Muscle Cells. PLoS ONE 9(4), e93508 (2014). 24690900 10.1371/journal.pone.0093508 PMC3972239

[b3] Robert J. Laumbach & Howard M. Kipen. Respiratory Health Effects of Air Pollution: Update on Biomass Smoke and Traffic Pollution. J Allergy Clin Immunol 129(1), 3–13 (2012). 22196520 10.1016/j.jaci.2011.11.021 PMC3272333

[b4] Hanne K. C. . Exposure to traffic and lung function in adults: a general population cohort study. BMJ Open 5, e007624 (2015). 10.1136/bmjopen-2015-007624 PMC4479998 26109116

[b5] Dominici F. . Fine particulate air pollution and hospital admission for cardiovascular and respiratory diseases. JAMA 295, 1127–34 (2006). 16522832 10.1001/jama.295.10.1127 PMC3543154

[b6] Mary B. R. . Long-Term Exposure to Traffic Emissions and Fine Particulate Matter and Lung Function Decline in the Framingham Heart Study. Am J Respir Crit Care Med 191(6), 656–664 (2015). 25590631 10.1164/rccm.201410-1875OC PMC4384780

[b7] Andersen Z. J. . Chronic Obstructive Pulmonary Disease and Long-Term Exposure to Traffic-related Air Pollution: A Cohort Study. Am J Respir Crit Care Med 183(4), 455–461 (2011). 20870755 10.1164/rccm.201006-0937OC

[b8] Wang Guohui, Rajagopalan Sanjay, Sun Qinghua & Zhang Kezhong. Real-world exposure of airborne particulate matter triggers oxidative stress in an animal model. Int J Physiol Pathophysiol Pharmacol 2(1), 64–68 (2010). 21383899 PMC3047275

[b9] Mehra D., Geraghty P. M., Hardigan A. A. & Foronjy R. A Comparison of the Inflammatory and Proteolytic Effects of Dung Biomass and Cigarette Smoke Exposure in the Lung. PLoS ONE 7(12), e52889 (2012). 23285217 10.1371/journal.pone.0052889 PMC3527613

[b10] Hu G. P. . Development and systematic oxidative stress of a rat model of chronic bronchitis and emphysema induced by biomass smoke. Experimental Lung Research 39, 229–240 (2013). 23682816 10.3109/01902148.2013.797521

[b11] Ramos C. . Increase of matrix metalloproteinases in wood smoke-induced lung emphysema in guinea pigs. Inhalation toxicology 21, 119–132 (2009). 18836920 10.1080/08958370802419145

[b12] Zin Walter A. . Eugenol attenuates pulmonary damage induced by diesel exhaust particles. Appl Physiol 112, 911–917 (2012). 10.1152/japplphysiol.00764.2011 22194320

[b13] Kalluri R. & Weinberg R. A. The basics of epithelial-mesenchymal transition. J Clin Invest 119(6), 1420–1428 (2009). 19487818 10.1172/JCI39104 PMC2689101

[b14] Willis B. C. & Borok Z. TGF-beta-induced EMT: mechanisms and implications for fibrotic lung disease. Am J Physiol Lung Cell Mol Physiol 293(3), L525–534 (2007). 17631612 10.1152/ajplung.00163.2007

[b15] Willis B. C. . Induction of epithelial- mesenchymal transition in alveolar epithelial cells by transforming growth factor-beta1: potential role in idiopathic pulmonary fibrosis. Am J Pathol 166, 1321–1332 (2005). 15855634 10.1016/s0002-9440(10)62351-6 PMC1606388

[b16] Zhang M. . TGF-beta1 induces human bronchial epithelial cell-to- mesenchymal transition *in vitro*. Lung 187, 187–194 (2009). 19252942 10.1007/s00408-009-9139-5

[b17] Gao W. . Bronchial epithelial cells: The key effector cells in the pathogenesis of chronic obstructive pulmonary disease? Respirology 20, 722–729 (2015). 25868842 10.1111/resp.12542

[b18] Sint T., Donohue J. F. & Ghio A. J. Ambient air pollution particles and the acute exacerbation of chronic obstructive pulmonary disease. Inhal Toxicol 20, 25–9 (2008). 18236218 10.1080/08958370701758759

[b19] Torres-Duque C., Maldonado D., Perez-Padilla R., Ezzati M. & Viegi G. Biomass fuels and respiratory diseases: a review of the evidence. Proc Am Thorac Soc 5, 577–90 (2008). 18625750 10.1513/pats.200707-100RP

[b20] Lopes F. D. . Exposure to ambient levels of particles emitted by traffic worsens emphysema in mice. Environ Res 109, 544–51 (2009). 19362299 10.1016/j.envres.2009.03.002

[b21] Zhou Y. . Lung function and incidence of chronic obstructive pulmonary disease after improved cooking fuels and kitchen ventilation: a 9-year prospective cohort study. PLoS Med 11(3), e1001621 (2014). 24667834 10.1371/journal.pmed.1001621 PMC3965383

[b22] Liu S. M. . Biomass fuels are the probable risk factor for chronic obstructive pulmonary disease in rural South China. Thorax 62, 889–897 (2007). 17483137 10.1136/thx.2006.061457 PMC2094241

[b23] Liu S. . Association between exposure to ambient particulate matter and chronic obstructive pulmonary disease: results from a cross-sectional study in China. Thorax 10.1136, thoraxjnl 208910 (2016). 10.1136/thoraxjnl-2016-208910 PMC5738534 27941160

[b24] Brito J. M. . Acute cardiovascular and infiammatory toxicity induced by inhalation of diesel and biodiesel exhaust particles. Toxicol. Sci 116, 67–78 (2010). 20385657 10.1093/toxsci/kfq107

[b25] Hemmingsen J. G., Moller P., Nojgaard J. K., Roursgaard M. & Loft S. Oxidative stress, genotoxicity, and vascular cell adhesion molecule expression in cells exposed to particulate matter from combustion of conventional diesel and methyl ester biodiesel blends. Environ. Sci. Technol 45, 8545–8551 (2011). 21842833 10.1021/es200956p

[b26] Tzamkiozis T. . Monitoring the infiammatory potential of exhaust particles from passenger cars in mice. Inhal. Toxicol 22 (Suppl. 2), 59–69 (2010). 21029033 10.3109/08958378.2010.519408

[b27] Mauderly J. L. . The National Environmental Respiratory Center (NERC) experiment in multi-pollutant air quality health research: II. Comparison of responses to diesel and gasoline engine exhausts, hardwood smoke and simulated downwind coal emissions. Inhal Toxicol. 26(11), 651–67 (2014). 25162719 10.3109/08958378.2014.925523

[b28] Sohal S. S. . Reticular basement membrane fragmentation and potential epithelial mesenchymal transition is exaggerated in the airways of smokers with chronic obstructive pulmonary disease. Respirology 15(6), 930–8 (2010). 20630030 10.1111/j.1440-1843.2010.01808.x

[b29] Câmara Joana & Jarai. Gabor Epithelial-mesenchymal transition in primary human bronchial epithelial cells is Smad dependent and enhanced by fibronectin and TNF-α. Fibrogenesis & Tissue Repair 3, 2 (2010). 20051102 10.1186/1755-1536-3-2 PMC2821296

[b30] Sukhwinder S. S. . Evaluation of epithelial mesenchymal transition in patients with chronic obstructive pulmonary disease. Respiratory Research 12, 130 (2011). 21970519 10.1186/1465-9921-12-130 PMC3198934

[b31] Groenewegen K. H. . Increased systemic inflammation is a risk factor for COPD exacerbations. Chest 133, 350–357 (2008). 18198263 10.1378/chest.07-1342

[b32] van Eeden S. F., Yeung A., Quinlam K. & Hogg J. C. Systemic response to ambient particulate matter: relevance to chronic obstructive pulmonary disease. Proc. Am. Thorac. Soc 2, 61–67 (2005). 16113470 10.1513/pats.200406-035MS

[b33] Peter J. Barnes. The cytokine network in asthma and chronic obstructive pulmonary disease. J. Clin. Invest. 118, 3546–3556 (2008). 18982161 10.1172/JCI36130 PMC2575722

[b34] Tuder Rubin M. & Petrache. Irina Pathogenesis of chronic obstructive pulmonary disease. J. Clin. Invest. 122(8), 2749–2755 (2012). 22850885 10.1172/JCI60324 PMC3408733

[b35] Motz Gregory T., Eppert Bryan L., Wesselkamper Scott C., Flury Jennifer L. & T. Michael Borchers. Chronic Cigarette Smoke Exposure Generates Pathogenic T Cells Capable of Driving COPD-like Disease in Rag2^-/-^ Mice. Am J Respir Crit Care Med 181, 1223–1233 (2010). 20133926 10.1164/rccm.200910-1485OC PMC2891493

[b36] Francesca P. . A Novel Nonhuman Primate Model of Cigarette Smoke Induced Airway Disease. The American Journal of Pathology 185(3), 741–755 (2015). 25542772 10.1016/j.ajpath.2014.11.006 PMC4348468

[b37] Mucha L., Stephenson J., Morandi N. & Dirani R. Meta-analysis of disease risk associated with smoking, by gender and intensity of smoking. Gend Med 3, 279e291 (2006). 17582369 10.1016/s1550-8579(06)80216-0

[b38] Li Z. T. . Bone morphogenetic protein 4 inhibits liposaccharide-induced inflammation in the airway. Eur. J. Immunol 44, 3283–3294 (2014). 25142202 10.1002/eji.201344287

