# Supplementary material for: Exposure to Ambient Particulate Matter Induced COPD in a Rat Model and a Description of the Underlying Mechanism
Source: Sci Rep. 2017 Mar 31;7:45666. doi: 10.1038/srep45666 (PMC5374504; doi:10.1038/srep45666)
Supplement: Supplemental Text [file srep45666-s1.pdf]

SUPPLEMENTAL INFORMATION

Exposure to Ambient Particulate Matter Induced COPD in a Rat Model and a Description of the Underlying Mechanism

Fang He, Baoling Liao, Jinding Pu, Chenglong Li, Mengning Zheng, Lingmei Huang, Yumin Zhou, Dongxing Zhao, Bing Li, Pixin Ran

Supplemental Results

Determination of Gas Concentrations in the Exposure Rooms During Air Pollution PM Exposure. We measured gas concentrations inside the chambers because gaseous co-pollutants are generated by combustion. The O_2, CO, NO_X and SO_2 levels in the exposure rooms during BMF and MVE exposure are shown in Fig. S1. The CO and NO_X levels were higher in the BMF group than in the MVE group, whereas the O_2 levels were not significantly different between the two groups. The SO_2 levels were low in both groups, and the SO_2 levels in the MVE group were slightly higher than those in the BMF group.

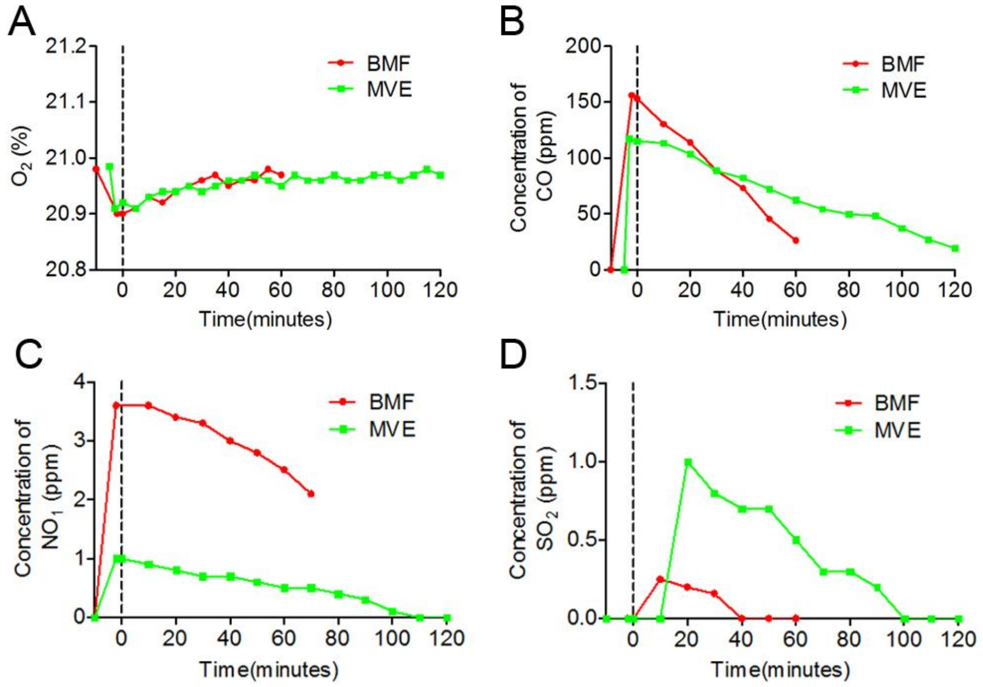

Figure S1. The O_2, CO, NO_X and SO_2 levels in the exposure rooms during air pollution PM exposure. A, B, C, D: The CO and NO_X levels were higher in the BMF group than those in the MVE group, whereas the O_2 levels were not significantly different between the two groups. The SO_2 levels were low in both groups, the SO_2 levels in the MVE group were slightly higher than those in the BMF group.

Effects of Air Pollution PM on Survival and Body Weight. Consistent with human patients

with COPD, we found that rats with COPD showed restless behavior, wheezing, and shortness of breath within the first 2 months of developing COPD. As COPD progressed to later stages, the rats showed progressive and gradual reductions in body weight. As shown in Fig. S2, there were no significant differences among the rats in all three groups in terms of initial body weight. From 4 months of exposure onward, the mean weight gain was lower in the BMF group and the MVE group than in the control group. Furthermore, there was a significant difference ($p<0.05$) between the two exposure groups and the control group regarding weight gain after 5 months of exposure. All of the rats were fed a standard diet throughout the experimental period. At the end of the experiment, after 7 months of exposure, the mean weight gain was 155.25±35.26 g in the control group, 102.15±32.23 g in the BMF group, and 100.21±22.64 g in the MVE group. These results show that the BMF- and MVE-exposed rats had attenuated rates of weight gain compared to the control group.

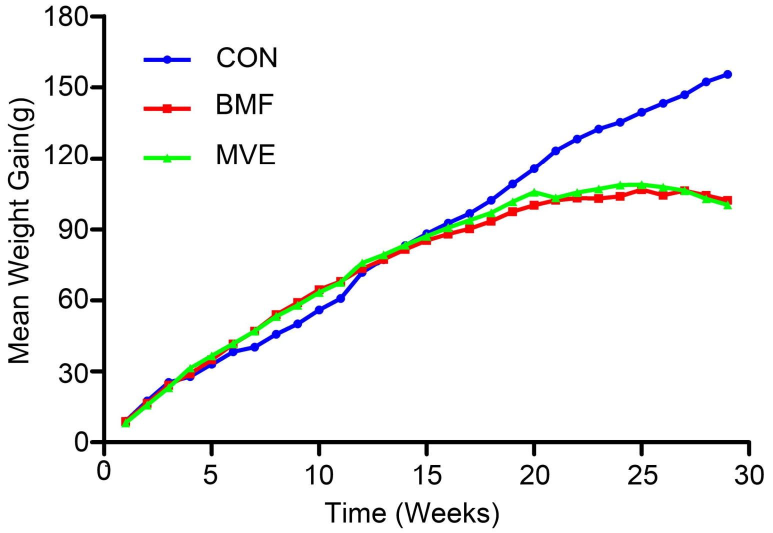

Figure S2. Effects of air pollution PM on body weight. The body weights (g) of the rats from the different study groups were measured from 1 week to 29 weeks (7 months) of the exposure period. Values are expressed as the mean weight gain. After 5 months of exposure,

the mean weight gain was lower in the BMF group and the MVE group than in the control group. There was a significant difference ($p< 0.05$) between the exposure group and the control group regarding weight gain (n=14-32). **Data are expressed as the mean±SEM** (A-D)**. *P < 0.05, **P < 0.01.**

Air pollution PM Exposure Induces Apoptosis of Alveolar Septal Cells, Leading to Airspace Enlargement. Walter A and colleague found diesel (DIE)-exposed animals showed higher levels of apoptosis in lung sections evaluated using TUNEL staining[S1]. In addition, Francesca found cigarette smoke increased alveolar septal cell apoptosis in NHPs[S2]. We evaluated airway apoptosis by detecting DNA fragmentation using TUNEL staining. The present study revealed that significant increases in DNA fragmentation counts were detected after 3 and 5 months of BMF or MVE exposure. The results indicated that the BMF exposure-induced alveolar septal cell apoptosis was more severe (Fig. S3). In addition to inflammation, apoptosis might be another important mechanism leading to alveolar enlargement in rats after air pollution PM exposure.

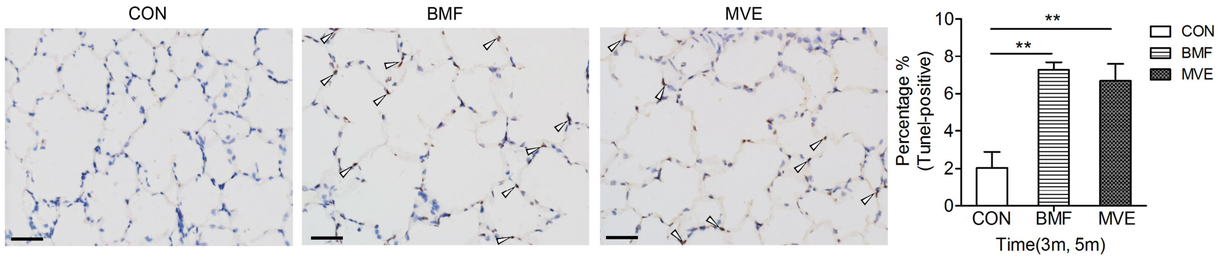

Figure S3. Air pollution PM exposure induces apoptosis of alveolar septal cells. TUNEL staining of lung tissues harvested from rats after 3 and 5 months of BMF or MVE exposure (n=8). Compared with controls, significant increases in TUNEL-positive cells were detected after 3 and 5 months of BMF or MVE exposure ($p< 0.01$). The TUNEL-positive cells in the BMF-exposed rats were higher than those in the MVE-exposed rats. The white arrow

indicates a TUNEL-positive cell. **Data are shown as the mean±SEM. *P < 0.05, **P < 0.01. Original magnification, x400.**

Supplemental Materials and Methods

Exposure System. PM mass concentrations and particle size distribution were measured using DustTraks (TSI 8533, USA) with a time resolution of 1 s[S3,S4]. A DustTrak has a flow rate of 1.7 LPM and measures mass concentrations of total suspended particles (PM total) as well as PM_{10}, $PM_{2.5}$, and PM in the range of 0.001–150 mg/m^3.

NO, SO_2, CO and O_2 concentrations were measured using an electrochemical gas analyzer (Testo 340, Germany)[S5]. This instrument was capable of measuring NO concentrations within a range of 0-3000 ppm, SO_2 concentrations within a range of 0-5000 ppm, CO concentrations within a range of 0-10,000 ppm, and O_2 concentrations within a range of 0-25%. The measurement accuracies for these four components were ±5%, ±5%, ±5% and ±1%. NOx and CO concentrations were corrected to a 0% O_2 level in the product stream, and the NOx concentration was measured as the sum of the amounts of NO and NO_2.

Measurement of the Apoptosis of Alveolar Septal Cells. Apoptotic cells in lung tissues were evaluated using Terminal-deoxynucleoitidyl transferase mediated nick end labeling (TUNEL) staining with a Roche *In Situ* Cell Death Detection Kit (Roche, U.S.) according to the manufacturer's protocol. The proportion of TUNEL-positive alveolar septal cells was estimated by an experienced histologist blinded to the treatment conditions. The quantitation of the TUNEL-positive cells is expressed as the percentage of total cells in the lung sections.

Bronchoalveolar Lavage. Rats were weighed, sacrificed via intraperitoneal injection of sodium pentobarbital (> 100 mg/kg) and exsanguinated. The trachea was cannulated with a

blunted 22-gauge needle, the left lung was lavaged 3 times, and bronchoalveolar lavage (BAL) was performed using cold, sterile, pyrogen-free, Ca^{2+} and Mg^{2+}-free phosphate-buffered saline (PBS) at a volume of 2.5 ml for the first lavage and 3 ml for subsequent lavages. Approximately 8 ml of BALF per rat was collected in sterile centrifuge tubes. Pooled BALF cells for each rat were washed in PBS followed by centrifugation (800 × g for 10 min at 4 °C). Cell-free first fractions of BALF aliquots were frozen at −80 °C for protein and cytokine evaluations[S6].

BALF Cell Counting and Differentials. Cells were collected from BALF through centrifugation, and the cell pellets were resuspended in 1 ml of PBS. A 10-µl aliquot of each sample was stained with trypan blue and analyzed using a hemocytometer to determine cell number and viability. PBS (100 µl) was then added to the cells, which were resuspended onto slides. After being fixed with 10% paraformaldehyde for 24 h, the slides were stained with hematoxylin and eosin (H&E) (Solarbio Tech; Beijing, China) and analyzed at x400 by light microscopy. A total of 400 cells per sample were counted to determine the percentages of alveolar macrophages, neutrophils and lymphocytes.

Sampling Lung Tissues and Blood. Rats were sacrificed by intraperitoneal injection of sodium pentobarbital (100 mg/kg). The left or right lungs were inflated and fixed using 4% paraformaldehyde (pH 7.40) at 25 cm H_2O pressure for 24 h. The lungs were then embedded in paraffin and cut into 4-µm-thick sections. The right lung tissues were snap frozen in liquid nitrogen and stored at -80°C for Western blot analysis. The body cavity was opened and blood samples were collected from the inferior vena cava; serum was stored at -80°C for ELISA.

Morphometric Measurements. To avoid observer bias, all microscope slides were coded

before analysis by one observer and were read blindly. Photographs were taken on a Zeiss Axio Imager 2 Microscope (Carl Zeiss, Germany), and morphometric analysis was performed using Image-Pro Plus (IPP) 7.0 software (Media Cybernetics, Silver Spring, USA). Airspace size was quantified in lung tissues stained with H&E using the mean linear intercept[S7,S8], and an algorithm was applied to perform quantitative characterization of airspace enlargement[S9]. The thickness of the small airway wall was analyzed according to previously described methods[S9,S10]. Small airways cut transversely and with a basement membrane perimeter (Pbm) of less than 2000 μm were examined. The results were standardized for airway size based on the Pbm ($\mu m^2/\mu m$). At least five small airways were counted per slide[S11]. Collagen deposition around small airways and vessels was assessed in paraffin-embedded and formalin-fixed lung sections stained with Masson's trichrome using a commercial kit (Sigma-Aldrich). The areas of collagen deposited around airways and vessels were quantified based on areas that were stained blue by Masson's trichrome using image analysis. Periodic acid-Schiff (PAS) staining is mainly used to visualize structures containing a high proportion of carbohydrate macromolecules, such as connective tissues, mucus, the glycocalyx, and basal laminae. Lung sections were stained with AB-PAS using commercial kits (Sigma-Aldrich, St. Louis, MO) following the manufacturer's instructions.

Immunohistochemistry and Immunofluorescence. Immunohistochemical (IHC) evaluation was performed as described elsewhere[S12]. The sections were incubated with primary antibodies against a-SMA (Sigma-Aldrich) or MUC5AC (Abcam; Cambridge, UK). A horseradish peroxidase (HRP)-conjugated secondary antibody was also used and visualized with diaminobenzidine using an Immunohistochemistry Detection Kit (Gene Tech; Shanghai,

China) according to the manufacturer's protocols. Photographs were taken using identical conditions for light setting and contrast. Color segmentation protocols in the IPP7.0 system were utilized to obtain data. The area of a-SMA-immunostaining in the small airway wall was quantitatively analyzed[S13]. Immunofluorescence evaluation was performed as described elsewhere[S14]. The tissue sections were then incubated with primary antibodies (against E-cadherin, vimentin, or FSP1) and detected using an appropriate fluorochrome-linked secondary antibody. DAPI was used as a nuclear counterstain. Images were acquired using a Zeiss Axio Imager 2 microscope (Carl Zeiss, Germany). Double immunofluorescence staining of E-cadherin (BD Biosciences; California, USA) and vimentin or FSP1 (Abcam; Cambridge, UK) was performed using a MultiVision polymer detection system (Thermo Scientific; Utah, USA) according to the manufacturer's protocols.

Western Blot Analysis. Levels of MMP9 and MMP2 proteins in the lung were evaluated by Western blotting as previously described[S15]. Lung tissues were ground into powder using liquid nitrogen in a mortar. Tissue lysates were collected into EP tubes and centrifuged at 10,000 rpm for 10 min at 4°C. The protein content in the supernatant was measured using a BCA Protein Assay Kit (Keygenbio, Nanjing). A total of 80 μg protein from each group of samples was assayed by SDS-PAGE. Following electrophoretic transfer, membranes were treated at RT for 1 hour with 5% skim milk. Then, the membranes were incubated overnight at 4°C with primary antibodies against MMP9, MMP2 (Abcam; Cambridge, UK), and GAPDH (Santa Cruz Biotechnology; California, USA). The membranes were then incubated with secondary antibodies conjugated to HRP (Santa Cruz Biotechnology; California, USA). Immunodetection was performed by chemiluminescence (ECL, Millipore). Relative protein

levels were quantified and normalized to GAPDH protein levels.

Measurement of Cytokines Using Bio-Plex. Cytokines in BALF and blood sera from rats exposed to BMF and MVE were analyzed using a Bio-Plex system (Bio-Rad, CA, USA). Using a rat cytokine 24-Plex assay kit (171-K1001 M), both BALF and blood sera were assayed for the following 24 cytokines: IL-1α, IL-1β, IL-2, IL-4, IL-5, IL-6, IL-7, IL-10, IL-12p70, IL-13, IL-17A, IL-18, EPO, M-CSF, G-CSF, GM-CSF, Gro/KC, INF-γ, MCP-1, MIP-1α, MIP-3α, RANTES, TNF-α, and VEGF. For each sample, 3-fold diluted aliquots of blood serum and BALF (80 μl taken as is) were used to analyze and estimate the concentrations of cytokines, chemokines and growth factors. All samples were measured in duplicate, and the average values were used. The concentrations were calculated using Bio-Plex Manager 6.1 software (Bio-Rad, Tokyo) based on standard curves[S6].

Measurement of TGF-β1 by ELISA. TGF-β1 levels in the sera of the rats exposed to air pollution PM for 7 months (BMF and MVE) were measured according to the instructions included in a TGF-β1 Rat ELISA kit (Abcam; Cambridge, UK). For each sample, a 250-fold diluted aliquot of serum was used to analyze and estimate the concentration of TGF-β1. All samples were measured in duplicate, and the average values were used. The concentrations were calculated using SkanIt Software 2.4.3 RE for Varioskan Flash (Thermo Scientific, Finland) based on standard curves.

Supplemental References

S1. Walter A. Zin, *et al.* Eugenol attenuates pulmonary damage induced by diesel exhaust particles. *Appl Physiol* **112,** 911–917 (2012).

S2. Francesca, P. *et al.* A Novel Nonhuman Primate Model of Cigarette Smoke Induced

Airway Disease. *The American Journal of Pathology* **185(3),** 741-755 (2015).

S3. Debra L. Laskin, Gedi Mainelis, Barbara Turpin, Kinal J. Patel, Vasanthi R. Sunil. Pulmonary Effects of Inhaled Diesel Exhaust in Young and Old Mice: A Pilot Project. *Res Rep Health Eff Inst* **151,** 3–31 (2010).

S4. Kwak JH, Kim H, Lee J, Lee S. Characterization of non-exhaust coarse and fine particles from on-road driving and laboratory measurements. *Sci Total Environ.***458-460**, 273-82 (2013).

S5. B Yu，SM Kum，CE Lee，S Lee. Effects of exhaust gas recirculation on the thermal efficiency and combustion characteristics for premixed combustion system. *Energy* **49(49)**, 375-383 (2013).

S6. Naveena, Y. *et al.* Biodiesel versus diesel exposure: Enhanced pulmonary inflammation, oxidative stress, and differential morphological changes in the mouse lung. *Toxicol Appl Pharmacol* **272(2)**, 373–383 (2013).

S7. Kelly, Y. *et al.* Chronic exposure of diesel exhaust particles induces alveolar enlargement in mice. *Respir Res* **16(1)**, 18 (2015).

S8. Takubo, Y. *et al.* Alpha-1 antitrypsin determines the pattern of emphysema and function in tobacco smoke-exposed mice: parallels with human diseases. *Am J Respir Crit Care Med* **166**, 1596–603 (2002).

S9. Bracke, K. R. *et al.* Cigarette smoke-induced pulmonary inflammation, but not airway remodeling, is attenuated in chemokine receptor 5-deficient mice. *Clin Exp Allergy* **37(10)**, 1467-79 (2007).

S10. Zou, Y. M. *et al.* Upregulation of Gelatinases and Epithelial–Mesenchymal Transition in

Small Airway Remodeling Associated with Chronic Exposure to Wood Smoke. *PLoS One* **9(5)**, e96708 (2014).

S11. Cho, J. Y. *et al.* Immunostimulatory DNA inhibits transforming growth factor-b expression and airway remodeling. *Am J Respir Cell Mol Biol* **30***,* 651–661 (2004).

S12. Selman, M. *et al*. Matrix metalloproteinases inhibition attenuates tobacco smoke-induced emphysema in guinea pigs. *CHEST Journal* **123**, 1633–1641 (2003).

S13. Kohan M, Breuer R, Berkman N. Osteopontin induces airway remodeling and lung fibroblast activation in a murine model of asthma. *Am J Respir Cell Mol Bio* **l41**, 290–296 (2009).

S14. March, T. H. *et al.* Modulators of cigarette smoke-induced pulmonary emphysema in A/J mice. *Toxicol Sci* **92**, 545-559 (2006).

S15. Qi, Y. *et al*. Inhibition of AMPK expression in skeletal muscle by systemic inflammation in COPD rats. *Respiratory Research* **15**, 156 (2014).
